# The Plantago Major in Spider Bite

**Published:** 1857-11

**Authors:** 


*The Plantago Major in Spider Bite.*—Dr. D. W. Maull in an article in the *Med. & Surg. Reporter* recommends the Plantago Major as an efficient remedy for the bite of the venomous spider. He says: "We are led to regard it almost in the light of a specific, if such a term is admissible." "The mode of preparing and admidistering is, to express the juice from the fresh leaves, and give three or four fluid ounces at a time."

